# Aldosterone synthase inhibitors: a new option for antihypertensive, cardio-renal protection

**DOI:** 10.1038/s41440-026-02671-z

**Published:** 2026-05-21

**Authors:** Atsuhisa Sato, Mitsuhiro Nishimoto

**Affiliations:** 1 Department of Internal Medicine, Division of Nephrology and Hypertension, International University of Health and Welfare School of Medicine, Yaita city, Tochigi Japan; 2 Department of Internal Medicine, Division of Nephrology, International University of Health and Welfare School of Medicine, Mita Hospital, Minato-ku, Tokyo Japan

**Keywords:** Aldosterone synthase inhibitor, Mineralocorticoid receptor antagonist, Hypertension, Chronic kidney disease, Cortisol.

## Abstract

Hypertension and organ damage caused by an inappropriate balance between aldosterone and salt are deeply involved in pathologies such as chronic heart failure, chronic kidney disease, and vascular disorders. Mineralocorticoid receptor antagonists are effective not only as antihypertensives, but also as organ-protective drugs, blocking direct aldosterone-induced organ damage independent of blood pressure. In recent years, research into selective aldosterone synthase inhibitors has progressed, resulting in the development of drugs such as baxdrostat, lorundrostat, and vicadrostat. These drugs are highly selective for aldosterone synthase with little inhibition of 11β-hydroxylase, making them promising antihypertensive and organ-protecting drugs. This article summarizes the basic and clinical research on aldosterone synthase inhibitors to date and reviews their characteristics. Aldosterone synthase inhibitors are drugs with the unprecedented characteristic of reducing blood aldosterone levels. Many studies have shown that low serum aldosterone levels are associated with a reduced risk of cardiovascular events. In fact, clinical trials of aldosterone synthase inhibitors have shown that lowering serum aldosterone levels by 50–70% results in lower blood pressure and organ protection. Even a small reduction in serum or local aldosterone levels is highly likely to improve hypertension and organ damage. Clinically, lowering blood aldosterone levels is very important.　Compared to the clinical evidence for mineralocorticoid receptor antagonists, clinical evidence for aldosterone synthase inhibitors remains insufficient. How these two drug types should be used given their differences thus remains unresolved. Discussing the differences in clinical effects in the future is thus important.

**Figure:**
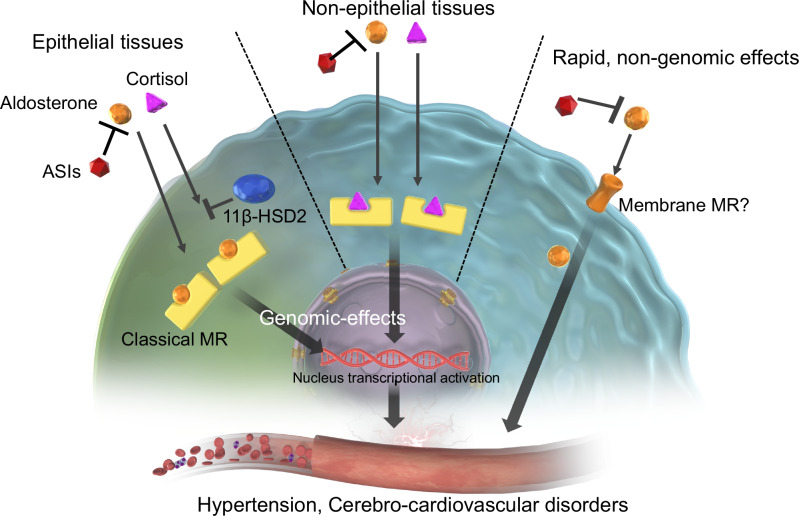
**The site of action of aldosterone synthase inhibitors (ASIs), and differences in mineralocorticoid receptors (MR)-mediated actions in epithelial and non-epithelial tissues.** Although the MR in epithelial and non-epithelial tissues are genetically identical, their physiological functions differ significantly. In epithelial tissues, aldosterone is the physiological ligand because cortisol is inactivated to cortisone by the enzyme 11β-hydroxysteroid dehydrogenase type 2 (11β-HSD2). In contrast, in non-epithelial tissues, this enzyme is absent or present at low levels, making cortisol, which has approximately 100-fold higher free circulating levels, the physiological ligand. The actions of aldosterone and cortisol are mediated by intracellular classical MR, but the existence of a putative membrane MR with high affinity exclusively for aldosterone is also thought to exist. Upon binding to the ligand, MR translocates to the nucleus, and acts through transcriptional activity. On the other hand, membrane MR is thought to mediate rapid, non-genomic effects that do not involve transcriptional activity. ASIs may attenuate aldosterone actions without significantly affecting cortisol actions in epithelial tissues. In non-epithelial tissues, aldosterone actions are not major, and ASIs do not affect the physiological actions of cortisol via MR. Further investigation is needed to determine the pharmacological effects of this mechanism, and how it differs from MR antagonists (The recruitment of co-activators and co-repressors in transcriptional activity is omitted)

**The site of action of aldosterone synthase inhibitors (ASIs), and differences in mineralocorticoid receptors (MR)-mediated actions in epithelial and non-epithelial tissues.** Although the MR in epithelial and non-epithelial tissues are genetically identical, their physiological functions differ significantly. In epithelial tissues, aldosterone is the physiological ligand because cortisol is inactivated to cortisone by the enzyme 11β-hydroxysteroid dehydrogenase type 2 (11β-HSD2). In contrast, in non-epithelial tissues, this enzyme is absent or present at low levels, making cortisol, which has approximately 100-fold higher free circulating levels, the physiological ligand. The actions of aldosterone and cortisol are mediated by intracellular classical MR, but the existence of a putative membrane MR with high affinity exclusively for aldosterone is also thought to exist. Upon binding to the ligand, MR translocates to the nucleus, and acts through transcriptional activity. On the other hand, membrane MR is thought to mediate rapid, non-genomic effects that do not involve transcriptional activity. ASIs may attenuate aldosterone actions without significantly affecting cortisol actions in epithelial tissues. In non-epithelial tissues, aldosterone actions are not major, and ASIs do not affect the physiological actions of cortisol via MR. Further investigation is needed to determine the pharmacological effects of this mechanism, and how it differs from MR antagonists (The recruitment of co-activators and co-repressors in transcriptional activity is omitted)

## Abstract (web summary)

The site of action of aldosterone synthase inhibitors (ASIs), and differences in mineralocorticoid receptors (MR)-mediated actions in epithelial and non-epithelial tissues. Although the MR in epithelial and non-epithelial tissues are genetically identical, their physiological functions differ significantly. In epithelial tissues, aldosterone is the physiological ligand because cortisol is inactivated to cortisone by the enzyme 11β-hydroxysteroid dehydrogenase type 2 (11β-HSD2). In contrast, in non-epithelial tissues, this enzyme is absent or present at low levels, making cortisol, which has approximately 100-fold higher free circulating levels, the physiological ligand. The actions of aldosterone and cortisol are mediated by intracellular classical MR, but the existence of a putative membrane MR with high affinity exclusively for aldosterone is also thought to exist. Upon binding to the ligand, MR translocates to the nucleus, and acts through transcriptional activity. On the other hand, membrane MR is thought to mediate rapid, non-genomic effects that do not involve transcriptional activity. ASIs may attenuate aldosterone actions without significantly affecting cortisol actions in epithelial tissues. In non-epithelial tissues, aldosterone actions are not major, and ASIs do not affect the physiological actions of cortisol via MR. Further investigation is needed to determine the pharmacological effects of this mechanism, and how it differs from MR antagonists (The recruitment of co-activators and co-repressors in transcriptional activity is omitted).

## Introduction

Hypertension and organ damage caused by an inappropriate balance between aldosterone and salt are deeply involved in pathologies such as chronic heart failure, chronic kidney disease (CKD), and vascular disorders [1–7]. Mineralocorticoid receptor (MR) antagonists are effective not only as antihypertensives, but also as organ-protective drugs, blocking direct aldosterone-induced organ damage independent of blood pressure [8, 9]. MR-mediated adverse effects are only observed in the presence of both aldosterone and salt. Laboratory studies have shown that even with high concentrations of aldosterone, complete elimination of salt from experimental systems prevents hypertension, inflammation, and fibrosis [10–12]. Therefore, while salt reduction is important, neutralizing the harmful effects of aldosterone is difficult unless salt intake is strictly limited. For example, some individuals with extremely low salt intake reportedly exhibit hyperaldosteronism without developing hypertension [13]. However, salt intake in those cases appears to have been so low that it is unfeasible in modern society and only possible under extremely controlled environments. Conversely, the fact that individuals can survive on such low salt intake shows the importance of the aldosterone/MR cascade in a salt-free environment. In such an environment, an enhanced renin-angiotensin-aldosterone system acts as a valuable salt-recycling system within the body.

Given these limitations of salt restriction, another major strategy is to lower aldosterone levels. Aldosterone is produced in the zona glomerulosa of the adrenal cortex, but the brain is known a site of aldosterone production outside the adrenal gland [14–16]. While production in other areas has been reported, the amounts are extremely low and not clinically significant [17]. Aldosterone synthesis within the brain is widely recognized, but occurs at levels orders of magnitude lower than that in the adrenal gland, and circulating aldosterone can be considered to be almost entirely synthesized by the adrenal gland. Aldosterone synthase, encoded by the CYP11B2 gene, synthesizes 18β-hydroxycorticosterone and then aldosterone from corticosterone. Meanwhile, 11β-hydroxylase, encoded by CYP11B1, synthesizes cortisol from deoxycortisol. These two enzymes share 94% amino acid identity [18, 19]. Drugs that inhibit aldosterone synthase therefore also suppress cortisol synthesis, making their development as therapeutic agents extremely difficult. Osilodrostat was initially developed as an aldosterone synthase inhibitor, but the cortisol-suppressing effect was eventually recognized in clinical practice, and this agent is instead currently used in pharmacotherapy for Cushing’s syndrome [20, 21].

In recent years, research into selective aldosterone synthase inhibitors has progressed, resulting in the development of drugs such as baxdrostat, lorundrostat, and vicadrostat. These drugs are highly selective for aldosterone synthase with little inhibition of 11β-hydroxylase, making them promising antihypertensive and organ-protecting drugs. This article summarizes the basic and clinical research on aldosterone synthase inhibitors to date and reviews their characteristics.

## Characteristics of epithelial and non-epithelial MR: what are the differences?

To understanding the characteristics of aldosterone synthase inhibitors, recognizing the differences from MR antagonists is important. A thorough understanding of the physiology and pathology of MR is thus required. MR belongs to the steroid/thyroid/retinoid/orphan receptor family and is a ligand-dependent transcription factor present in the cytoplasm. MR shares over 90% homology in the DNA-binding domain and over 50% homology in the ligand-binding domain with glucocorticoid receptor (GR), progesterone receptor (PR), and androgen receptor (AR), forming a subfamily [22]. However, what distinguishes MR from other steroid receptors is its binding to two distinct physiological ligands, aldosterone and glucocorticoids, and the clearly restricted localization, with very low expression levels in general [23, 24]. This contrasts with GR, which is widely expressed and abundant [25]. MR is present not only in epithelial tissues, which are responsible for electrolyte regulation, but also in non-epithelial tissues [23]. For example, the hippocampus, is the center of memory, and exhibits abundant MR expression, exceeding that of epithelial tissues.

Cortisol shows higher receptor affinity for MR than for GR [26]. In theory, cortisol first binds to the MR, saturates this binding, then binds to the GR. Although blood levels of cortisol are more than 1000 times higher than those of aldosterone, cortisol is approximately 95% bound to plasma proteins, resulting in approximately 100-fold greater free levels. However, this binding mode and subsequent transcriptional activity differ significantly between epithelial and non-epithelial tissues. Epithelial tissues contain the enzyme 11β-hydroxysteroid dehydrogenase type 2 (11β-HSD2) [27–30]. At the pre-receptor level, cortisol is inactivated by 11β-HSD2 to form cortisone, which shows lower affinity for MR and GR, resulting in a 10:1 intracellular ratio [31]. Even at this low concentration difference, glucocorticoids primarily bind to the MR, but the effects of aldosterone are evident in epithelial tissues. The action of 11β-HSD2, results in the consumption of nicotinamide adenine dinucleotide (NAD) as a coenzyme, resulting in the accumulation of the reduced form, NADH. This accumulated NADH has been hypothesized to affect the glucocorticoid/MR complex in epithelial tissues [31].

MRs are also present in non-epithelial tissues where 11β-HSD2 expression is absent or present at only low levels. These MRs are directly affected by hormone levels, and even at nadir glucocorticoid levels, more than 90% of the receptors are thought to be occupied by glucocorticoids [32, 33]. In epithelial tissues, when 11β-HSD2 activity is inhibited by licorice consumption, glucocorticoids are able to bind to the MR, and act as an MR agonist, mimicking aldosterone. In patients with apparent mineralocorticoid excess, where mutations in the gene for 11β-HSD2 cause enzyme deficiency or impairment, cortisol binds to epithelial MRs and acts as an MR agonist, mimicking aldosterone, and resulting in hypertension and hypokalemia [34]. In non-epithelial tissues, glucocorticoids act in an MR-silent manner in some circumstances and as MR antagonists in others, modulating the action of aldosterone. Further, MR in non-epithelial tissues is thought to function as a high-affinity corticosteroid receptor. For example, in central regions such as the brain, MR is largely unprotected, and cortisol represents the physiological ligand, mediating effects such as hemodynamics, cognition, and stress responses in the brain [35, 36]. Genetically, MR in epithelial and non-epithelial tissues are identical, but in physiological terms, the two tissues differ markedly. As a high-affinity corticosteroid receptor, MR in non-epithelial tissues plays a major role in the physiological actions of cortisol. Aldosterone synthase inhibitors do not block the cortisol/MR cascade. This represents a key feature, but understanding the physiological functions of cortisol/MR in epithelial and non-epithelial tissues is therefore important.

## Importance of lowering blood aldosterone levels

Spironolactone, is an MR antagonist, that was developed and put into clinical use in the 1960s. Eplerenone, a second-generation MR antagonist with enhanced MR selectivity, is available for use in Japan for the treatment of hypertension and chronic heart failure. Eplerenone is also used overseas. In the United States, it was approved in 2002 and is indicated for hypertension and heart failure after acute myocardial infarction. In Europe, it was first approved in the Netherlands in 2004 and is currently indicated for heart failure after myocardial infarction and chronic heart failure. It is not indicated for hypertension.　New types of MR antagonists that do not have a steroid skeleton are now available [8, 9]. MR antagonists are drugs that block MR at the receptor level, regardless of the ligand. On the other hand, the greatest feature of aldosterone synthase inhibitors is that they can reduce blood aldosterone concentrations and attenuate only the aldosterone/MR cascade.

Lowering circulating aldosterone levels may be of considerable clinical significance. Conversely, numerous studies have demonstrated that even a slight increase in aldosterone concentration can lead to increased MR occupancy, potentially contributing to organ damage. To assess aldosterone activation on the MRs of cardiomyocytes, Qin et al. generated transgenic mice overexpressing 11β-HSD2 in cardiomyocytes using the mouse α-myosin heavy chain promoter [37]. Overexpression of this enzyme converts glucocorticoids to receptor-inactive metabolites, allowing aldosterone to occupy the cardiomyocyte MR. These transgenic mice spontaneously developed cardiac hypertrophy, fibrosis, and heart failure, and died prematurely on a normal salt diet. Such studies confirmed the deleterious consequences of inappropriate activation of the cardiomyocyte MR by aldosterone and demonstrated that under physiological conditions, glucocorticoids exert a sustained inhibitory effect to prevent these consequences. Even a slight increase in aldosterone occupancy on the cardiomyocyte MR has been demonstrated to have deleterious effects. Garnier et al. generated transgenic mice overexpressing aldosterone synthase in the heart by gene targeting using the α-myosin heavy chain promoter. Basal coronary blood flow was reduced in isolated perfused hearts, and transgenic mice exhibited reduced vasodilation in response to acetylcholine, bradykinin, and sodium nitroprusside compared with wild-type mice, resulting in impaired coronary artery function [38]. Increased production of cardiac aldosterone caused significant endothelial-independent dysfunction of the coronary artery.

Clinically, the aldosterone breakthrough phenomenon indicates the risk of even a slight increase in blood aldosterone levels. In patients receiving angiotensin-converting enzyme inhibitors (ACE-Is), angiotensin receptor blockers (ARBs), or direct renin inhibitors, plasma aldosterone levels are suppressed shortly after treatment, but return to baseline levels within 6–12 months. With continued treatment, plasma aldosterone levels rise above baseline levels, referred to as the aldosterone breakthrough phenomenon [39–43]. These elevated levels are by no means as high as those seen in primary aldosteronism. Although it has risen compared to the previous value, plasma aldosterone levels after the breakthrough is now within the so-called normal range [43]. Even a slight increase in plasma aldosterone level above baseline can negate the organ-protective effects of ACE-Is or ARBs [39–42].

In patients with essential hypertension, significant correlations have been reported between blood aldosterone levels and reduced systemic arterial compliance [44], left ventricular mass [45], and markers of left ventricular dysfunction [46, 47]. Compared to patients with essential hypertension matched for age, sex, blood pressure, and duration of hypertension, patients with primary aldosteronism reportedly exhibit reduced myocardial perfusion [48, 49], more severe left ventricular hypertrophy, and diastolic dysfunction [50, 51]. Even at comparable blood pressures, patients with primary aldosteronism experience significantly more cardiovascular events [52]. Although blood aldosterone concentrations in patients with primary aldosteronism are not always significantly elevated above the normal range, levels are generally higher than those in patients with essential hypertension. High blood aldosterone concentrations are associated with greater organ damage than in patients with essential hypertension. Stowasser et al. compared normotensive individuals genetically diagnosed with familial hyperaldosteronism type I (FH-I) with age- and sex-matched normotensive individuals in terms of blood pressure, biochemical parameters, pulse wave velocity, and echocardiographic findings. Patients with FH-I showed evidence of concentric remodeling, including a thickened septum and posterior wall, a lower early peak mitral velocity, and a lower early-to-late peak ratio of diastolic mitral flow velocity. Aldosterone excess, even in the absence of hypertension, is associated with increased left ventricular wall thickness and impaired diastolic function [53]. A subgroup of essential hypertensive patients, including some who do not meet the diagnostic criteria for primary aldosteronism, reportedly exhibit a lack of susceptibility to aldosterone suppression under salt loading, and this subgroup is frequent [54]. These patients are likely to have mild aldosterone hypersecretion. Lowering circulating levels of aldosterone by administering aldosterone synthase inhibitors may facilitate blood pressure control.

There is a concept called borderline aldosteronism, which encompasses hypertensive patients who show a mild elevation in serum aldosterone levels, even if their screening for primary aldosteronism is negative or borderline, and patients who are screening positive for primary aldosteronism but have a negative confirmatory test [55]. Sartoli et al. defined hypertensive patients who were positive on screening tests and negative on confirmatory diagnostic tests as having aldosterone-associated hypertension [56]. During a 22-month follow-up period, they found that a similar number of patients with aldosterone-associated hypertension to that of patients with definitive primary aldosteronism developed resistant hypertension.　Shibata et al. defined hypertensive patients with hyperaldosteronemia that do not meet the diagnostic criteria for primary aldosteronism as a subtype of mineralocorticoid receptor-related hypertension, likely due to pathophysiological activation of the aldosterone-MR axis [57]. Although hypertensive patients who show borderline values for primary aldosteronism are occasionally seen on screening tests, these patients usually do not proceed to confirmatory diagnostic tests. Long-term exposure to mildly elevated aldosterone levels is thought to increase the risk of cardiovascular complications. In such patients, treatment to lower blood aldosterone levels is likely to be effective given the patient’s condition.

## Receptor or ligand? characteristics of aldosterone synthase inhibitors

Compared to the clinical evidence for MR antagonists, clinical evidence for aldosterone synthase inhibitors remains insufficient. How these two drug types should be used given their differences thus remains unresolved. Discussing the differences in clinical effects in the future is thus important. Here, we introduce the characteristics of aldosterone synthase inhibitors, but the clinical effects resulting from these characteristics and how they differ from those of MR antagonists remain unclear.

### Aldosterone synthase inhibitors do not block physiological effects of cortisol/MR

This represents greatest feature of aldosterone synthase inhibitors. Because blockade does not occur at the receptor level, attenuating only the action of the aldosterone/MR is theoretically possible. The MR selectivity of aldosterone is preserved in epithelial tissues such as renal tubules. MR antagonists inhibit the action of aldosterone and act as potassium-sparing diuretics. When aldosterone synthase inhibitors are used, the aldosterone/MR action is attenuated, but does this enhance the cortisol/MR cascade? Clinical data on aldosterone synthase inhibitors suggest this is not the case. If the cortisol/MR cascade were to be increased, sodium resorption would increase, making blood pressure less likely to decrease, and cortisol-mediated potassium excretion would increase, preventing an increase in serum potassium levels. In reality, aldosterone synthase inhibitors have demonstrated stable antihypertensive effects, with a tendency toward increased serum potassium levels. In epithelial tissues, only the aldosterone/MR system may be attenuated.

On the other hand, non-epithelial tissues, such as the brain, heart, and vascular walls, are more complex. MRs in non-epithelial tissues are thought to function as high-affinity corticosteroid receptors, with cortisol as the physiological ligand, mediating effects such as hemodynamics, cognition, and brain stress responses [35, 36]. The effects of corticosteroids on memory have been controversial. Studies in rats and chickens suggest that activation of MRs is essential for the storage of sensory memories, while normal levels of GR activation (in addition to pre-activated MRs) are essential for memory consolidation and retrieval [58, 59]. Tytherleigh et al. used a repeated-measures approach to investigate the effects of activating MRs alone, GRs alone, and both MRs and GRs on episodic and semantic components of working and declarative memory in patients with Addison’s disease [60]. MRs and GRs were activated with exogenous steroids specific for MR (9α-fluorohydrocortisone) or GR (dexamethasone), respectively. Participants performed better on a digit-back task when both receptors were activated compared to when only GRs were activated. They also performed better on Hopkins Verbal Learning Test recall when both receptors were activated compared to when only MRs or GRs were activated. The significance of such physiological protection is unclear.

### Blood aldosterone levels do not increase after treatment

MR antagonists are known to increase plasma aldosterone levels one month after treatment [61]. Using a receptor antagonist, the blocked ligand increases, causing the drug and increased ligand to compete for the receptor. If the effect of the drug wears off because the patients forget to take doses, a backlash can therefore be expected. Continuing to take receptor antagonists with a long duration of effect and strong binding to the receptor is thus important. On the other hand, if ligand levels are lowered, this competition is weakened.

### Effects on rapid, non-genomic actions of aldosterone

Aldosterone has been reported to have both genomic effects, which require a certain amount of time due to transcriptional activation, and rapid non-genomic effects, which mediate a rapid response [62, 63] (Table 1). While the rapid non-genomic effects of aldosterone are widely acknowledged, their physiological significance and potential importance in hypertension remain unclear. Limited studies suggest that the rapid non-genomic effects of aldosterone have been observed in both vascular smooth muscle cells and cardiomyocytes, so its role in instantaneous blood pressure regulation and other processes. The potential relevance of such effects to hypertension is clearly an area that needs investigation, but to date, there has been little to no research directly related to this area.

**Table 1:** Genomic and rapid non-genomic effects of aldosterone

		genomic effects	rapid non-genomic effects
Receptors	intervening receptors	classical MR	membrane MR (putative)
	location	intracellular	cell membrane
	year of cDNA cloning	1987	not yet
	enzymatic protection of aldosterpne-MR selectivity (epithelial tissues)	protected by 11β-HSD2	not protected by 11β-HSD2
	affinity for glucocorticoid	equivalent to aldosterone	high affinity only for aldosterone
Actions	time until effect appears	it takes a certain amount of time (because it is mediated by transcriptional activity)	extremely fast (because it does not involve transcriptional activity)
	by MR antagonist	suppressed	not suppressed (there are also reports that it can be suppressed)
	by actinomycin D, cycloheximide	suppressed	not suppressed

This document summarizes the differences between genomic effects and rapid, non-genomic effects, focusing on the mediating receptors and their actions. The detailed characteristics of rapid non-genomic effects are still not fully understood, and it is possible that new findings will be added or existing findings will be revised in future research

*MR* mineralocorticoid receptors, *11β-HSD2* 11β-hydroxysteroid dehydrogenase type 2

Rapid non-genomic effects have been thought to be mediated by membrane receptors present on the cell membrane, separate from the classical MR within the cell. However, the relevant receptor remains unidentified for now. The rapid effects of estrogen have been reported to be mediated, at least in part, through GPER/GPR30 (G protein-coupled estrogen receptor/G protein-coupled receptor 30) [64, 65]. Gros et al. demonstrated that aldosterone induces apoptosis via both GPER/GPR30 and MR, activating intermediate signaling pathways such as phosphatidylinositol 3-kinase, extracellular signal-regulated kinase, and myosin light chain phosphorylation [66]. Further, eplerenone has been shown to act as both an MR antagonist and a partial GPER/GPR30 antagonist. On the other hand, some studies have suggested that most of the rapid, non-genomic effects of aldosterone are mediated by classical MR, with a different time frame from the genomic effects [67, 68]. How MR antagonists and aldosterone synthase inhibitors differ in their effects on rapid, non-genomic effects remains unclear.

## Clinical results for aldosterone synthase inhibitors

Here, we introduce baxdrostat, lorundrostat, and vicadrostat, as drugs for which numerous clinical studies have been published (Table 2). Evidence is expected to emerge for their potential as antihypertensive and organ-protective drugs for the treatment of chronic kidney disease and chronic heart failure.

**Table 2:** Principal characteristics of three representative aldosterone synthase inhibitors

	Baxdrostat	Lorundrostat	Vicadrostat
Selectivity CYP11B2/CYP11B1	100	374	250
Plasma half-life (h)	～30	8～10	4.4–6.3
Aldosterone suppression (%)	51–85	>70	37–60
Clinical doses (mg)	0.5–8	12.5–100	3, 10, 20 (with or without empagliflozin 10 mg)
Drug metabolism	Liver	Not specified	BI 689875, a glucuronide metabolite of vicadrostat, is excreted
	10–30% is excreted unchanged in urine		primarily in urine despite being produced in the liver
Risk of hyperkalemia	yes	yes	yes
Potential future indications	Hypertension, Resistant hypertension, Primary aldosteronism	Hypertension, Resistant hypertension	Chronic kidney disease, HFmrHF/HFpEF
Representative clinical studies	BrigHTN (2023), BaxHTN (2025), SPARK-PA (2025)	Target-HTN (2023), Advance HTN (2025)	EASi-KIDNEY (2023), EASi-HF (on going)
	Bax24 (2025)	Launch-HTN (2025)	
Expected schedule for market launch in Japan	2027–2028	Not disclosed	After 2030

While there has been no announcement regarding the future schedule for these three drugs until their market launch, we have calculated a rough estimate of the typical timeline from the completion of a phase 3 trial to approval. Baxdrostat is already in the FDA’s priority review in the US and could be launched as early as 2026. In Japan, it is estimated to be around 2027–2028. Lorundrostat is expected to be launched around 2027 in the US, but its development in Japan has not been disclosed. Vicadrostat targets chronic heart failure or CKD, and a phase 3 trial is underway, but because it is an outcomes trial with a long follow-up period, it is expected to be launched after 2030.

## Baxdrostat

Baxdrostat is a potent, long-acting, selective antihypertensive agent with a 100:1 specificity compared to CYP11B1 and a plasma half-life of more than 30 h [69, 70]. Among healthy normotensive individuals, doses of 0.5–5 mg reduced aldosterone levels by 51–73% without affecting cortisol [71]. A phase 1 study in which patients were divided into three groups based on the degree of renal dysfunction reported no significant differences in the pharmacokinetic parameters of baxdrostat between groups, with no dose adjustment required in CKD patients [72]. The BrigHTN phase 2 study examined treatment-resistant hypertension [73], comparing the effects of baxdrostat at 0.5 mg, 1 mg, or 2 mg with those of placebo. The primary endpoint was the change in systolic blood pressure after 12 weeks. Baxdrostat significantly reduced blood pressure in a dose-dependent manner. Hyperkalemia ( > 6.0 mEq/L) was observed in two patients, but improved upon discontinuation of oral administration. In the BrigHTN trial, plasma aldosterone concentration was reduced at all doses used. However, only the high dose significantly reduced blood pressure. Gomez-Sanchez et al. speculated that high-dose baxdrostat may cross the blood-brain barrier and inhibit local aldosterone synthesis in the brain, thereby antagonizing MR in blood pressure-related centers, particularly the periventricular cerebral cortex [74].

A phase 3 trial of baxdrostat in patients with uncontrolled or treatment-resistant hypertension has been reported [75]. Enrolled patients had a sitting systolic blood pressure of 140 mmHg or greater but less than 170 mmHg despite stable treatment with two antihypertensive drugs (uncontrolled hypertension) or three or more antihypertensive drugs, including a diuretic (treatment-resistant hypertension). After a 2-week placebo run-in period, patients with a sitting systolic blood pressure of 135 mmHg or greater were randomly assigned in a 1:1:1 ratio to receive baxdrostat at 1 or 2 mg, or placebo once daily for 12 weeks. The primary endpoint was the change in sitting systolic blood pressure from baseline to week 12. A total of 796 patients were randomized, with 794 patients receiving 1 mg of baxdrosttat (*n* = 264), 2 mg of baxdrostat (*n* = 266), or placebo (*n* = 264) in addition to baseline therapy. The placebo-adjusted difference in sitting systolic blood pressure at week 12 was -8.7 mmHg in the baxdrostat 1 mg group and –9.8 mmHg in the baxdrostat 2 mg group (P < 0.001 for both comparisons). Hyperkalemia ( > 6.0 mEq/L) was reported in 6 patients (2.3%) in the 1 mg baxdrostat group, 8 patients (3.0%) in the 2 mg baxdrostat group, and 1 patient (0.4%) in the placebo group. In patients with uncontrolled or treatment-resistant hypertension, the addition of baxdrostat to background therapy resulted in a significantly lower sitting systolic blood pressure at week 12 than in the placebo group.

The Spark-PA study was a phase 2a trial for patients with primary aldosteronism [76]. Although the trial was very limited to 15 cases, baxdrostat resolved or reduced the severity of hypertension, excessive aldosterone production, and hypokalemia. Primary aldosteronism is considered the best indication for aldosterone synthase inhibitors, but further investigation is needed, including into the type of disease (adenoma or hyperplasia, uni- or bilateral, etc.).

## Lorundrostat

This drug shows specificity of 374:1 compared with CYP11B1 and a selective aldosterone synthase inhibitor with a plasma half-life of 8–10 h [69, 70]. The efficacy and safety of five doses of lorundrostat (12.5 mg, 50 mg, or 100 mg once daily and 12.5 mg or 25 mg twice daily) were compared with placebo in 167 patients with uncontrolled hypertension, low renin and high aldosterone (Target-HTN study[77];). Lorundrostat at 50 mg or 100 mg once daily significantly reduced blood pressure after 8 weeks. Plasma aldosterone concentrations were decreased in all treatment groups. Hyperkalemia was observed in 3.6% of participants. A similar reduction in blood pressure was also observed in patients with unsuppressed plasma renin activity.

The Launch-HTN study evaluated the blood pressure-lowering efficacy and safety of lorundrostat when added to a regimen of 2–5 antihypertensive agents in patients with uncontrolled, treatment-resistant hypertension [78]. Participants were randomized to receive lorundrostat at 50 mg/day for 6 weeks followed by 100 mg/day for 6 weeks, lorundrostat at 50 mg/day for 12 weeks, or placebo for 12 weeks. The primary endpoint was the change in automated systolic blood pressure at week 6 in participants randomized to receive lorundrostat 50 mg or placebo. For the combined lorundrostat 50 mg group, the least-squares mean change in automated office systolic blood pressure at week 6 was −16.9 mmHg compared to −7.9 mmHg for the placebo group (least-squares mean difference, −9.1 mmHg; P < 0.001). Hyponatremia, hyperkalemia, and decreased renal function were reported more frequently in the lorundrostat group compared with the placebo group. The antihypertensive efficacy and safety of lorundrostat were demonstrated in uncontrolled adult hypertension, including treatment-resistant hypertension.

In the Advance-HTN study, participants taking 2–5 antihypertensive agents and with a visit blood pressure of ≧140/90 mmHg were randomized to a 3-week standardized regimen of antihypertensive treatment. Participants with a 24-h mean blood pressure ≧130/80 mmHg (n = 285) were then randomized to receive placebo, a stable dose of lorundrostat 50 mg/day (stable dose group), or lorundrostat starting at 50 mg/day and increasing to 100 mg/day if systolic blood pressure reached ≧130 mmHg after 4 weeks (dose-adjusted group). The primary endpoint was the change in 24-h mean systolic blood pressure from baseline to week 12 in each lorundrostat group, assessed as the least-squares mean difference (placebo-adjusted change) from placebo [79]. After 12 weeks, the least-squares mean 24-h mean systolic blood pressure was −15.4 mmHg in the stable dose group, −13.9 mmHg in the dose-adjusted group, and −7.4 mmHg in the placebo group. The placebo-adjusted change in blood pressure was −7.9 mmHg in the stable dose group and −6.5 mmHg in the dose-adjusted group. The placebo-adjusted change in 24-h mean systolic blood pressure from baseline to week 4 in the lorundrostat combination group was −5.3 mmHg. Potassium levels ≧6.0 mmol/L were observed in five patients (5%) in the stable dose group, seven patients (7%) in the dose-adjusted group, and none in the placebo group. Lorundrostat was associated with a greater reduction in 24-h mean blood pressure compared with placebo in patients with uncontrolled, treatment-resistant hypertension.

## Vicadrostat

Vicadrostat is a highly selective aldosterone synthase inhibitor being developed for the reduction of chronic kidney disease and cardiometabolic risk [69, 70]. A phase 2 trial has been published in patients with chronic kidney disease (estimated glomerular filtration rate; 30–90 ml/min/1.73 m², urine albumin-to-creatinine ratio; 200-5000 mg/g Cr) treated with an ACE-I or ARB [80]. Patients were initially assigned to receive either the sodium-glucose transporter 2 inhibitor empagliflozin or placebo for 8 weeks, followed by 14 weeks of treatment with three doses of vicadrostat (3, 10, or 20 mg) or placebo. The primary endpoint was the change from baseline in albuminuria. Vicadrostat monotherapy reduced albuminuria by up to 39% compared with placebo, and combination therapy with empagliflozin reduced albuminuria by up to 46%, suggesting an additive effect. Systolic blood pressure also decreased slightly (4–6 mmHg), and the decrease was amplified (7–8 mmHg) when combined with empagliflozin. High-dose vicadrostat administration increased serum potassium levels (by 0.25–0.33 mEq/L from baseline, but 86% of patients with hyperkalemia were able to continue treatment without any intervention). Concomitant use of empagliflozin helped reduce hyperkalemia. Cortisol levels were largely unaffected, but adrenal insufficiency was reported in 7 of 436 patients (2%) in the vicadrostat group and 1 of 147 patients (1%) in the placebo group.

Given these promising findings, the EASi-KIDNEY multicenter, international, randomized, double-blind, placebo-controlled phase 3 trial (Clinical Trials. gov ID:NCT06531824) will enroll approximately 11,000 patients with chronic kidney disease to evaluate the long-term ability of vicadrostat, when added to standard of care (including an SGLT2 inhibitor), to reduce kidney disease progression, heart failure hospitalization, and cardiovascular mortality [81]. Another double-blind, randomized, parallel-group superiority trial, the phase 3 EASi-HF trial (ClinicalTrials.gov ID; NCT06424288), will evaluate the time to first occurrence of cardiovascular death or heart failure hospitalization with vicadrostat plus empagliflozin in approximately 6,000 patients with heart failure and a left ventricular ejection fraction ≧40%.

## Conclusion

Treatment to suppress hypertension and organ damage caused by an inappropriate balance of salt and aldosterone is extremely important in terms of preventing the onset of cerebrovascular and cardiovascular diseases. MR antagonists have been pioneers in disrupting this cascade, and have shown considerable clinical evidence. Now, a new drug with a novel mechanism of action is emerging. Aldosterone synthase inhibitors are drugs with the unique characteristic of lowering blood aldosterone levels. The drugs described in this paper have completed or are in the process of phase 3 trials, and their clinical efficacy is anticipated. Future challenges include elucidating their detailed mechanisms, as the mechanism of action of aldosterone synthase inhibitors is more complex than expected. It is of great interest to determine which effects are linked to which clinical effects. Furthermore, there are still many questions to be answered, such as how to differentiate their use from MR antagonists, or whether combination therapy is possible.
